# Neurophysiological Effects Associated With Subliminal Conditioning of Appetite Motivations

**DOI:** 10.3389/fpsyg.2019.00457

**Published:** 2019-03-05

**Authors:** Micah Amd, Sylvain Baillet

**Affiliations:** ^1^McConnell Brain Imaging Center, Montreal Neurological Institute, McGill University, Montreal, QC, Canada; ^2^Laboratory of Human Behavior Studies, Department of Psychology, Federal University of São Carlos, São Carlos, Brazil; ^3^Department of Psychology, School of Social Sciences, University of the South Pacific, Suva, Fiji

**Keywords:** subliminal conditioning, appetite, eating disorders, motivation, learning theory, magnetoencephalography

## Abstract

When attempting to encourage eating, explicitly providing statements like “eating is pleasant” may produce little effect. This may be due to subjective, negatively-valenced narratives evoked by perception of the verb “eating” (e.g., *eating* →*fat* →*lonely*), overriding any explicitly provided *eating-pleasant* valence information. In our study, we presented eating-related verbs under subliminal visual conditions to mitigate the onset of *eating*-associated deliberation. Verbs were linked with neutral or positively valenced terms across independent blocks. Modulations of event-related magnetoencephalographic (MEG) components and parietal activations in the alpha range (8–12 Hz) illustrated a significant effect of valence during pre-lexical time windows. We found significantly greater saliva production and declarations of increasing hunger after eating-related verbs were linked with positive terms. Orally reported preferences did not vary between conditions.

## Introduction

In classical conditioning, associating a minimally salient (neutral) stimulus with emotionally salient events [unconditioned stimuli (US)] can transform the former’s hedonic valences, making it a conditioned stimulus (CS; Staats and Staats, 1957; Mowrer, 1980). The establishment of valenced CS following systematic CS–US correlations is ubiquitous to human language. Propositions can function as context-specific “conditioning devices” due to their ability to transform the *valence* of the terms qualified (Mowrer, 1980). Within this framework, the proposition “eating is good” is conceptualized as a CS–US relation, where the positive valences from *good* (US) can transform the valences of *eating*-associated representations (Mowrer, 1980, pp. 112–116). Positively transforming the valences of *eating* (CS) can enhance appetite motivation given that valence is intrinsically linked to a motivational state (Peterson, 2002; Custers and Aarts, 2010).

**FIGURE 1:**
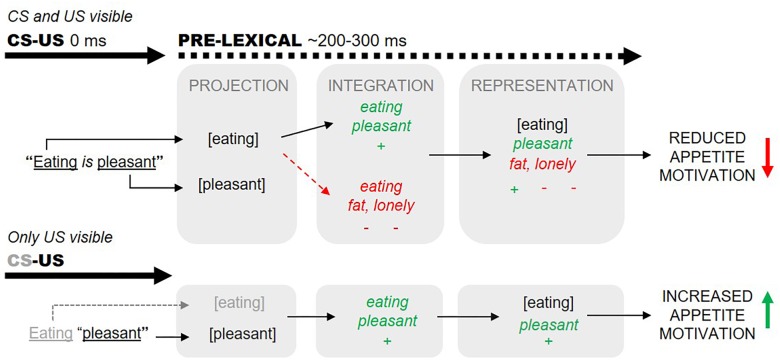
Valences predicated through the proposition “eating is pleasant” can be countered by subjective narratives evoked by conscious projection of the word “eating” (dashed arrows, top panel). Subjective and explicit valences are assumed to integrate within 200–300 ms of word onsets (before lexical processing). We aimed to minimize subjective eating-associated narratives by presenting “eating”-related words subliminally (bottom panel).

In practice, however, such clear-cut US-to-CS valence transformations are not always observed. We conjecture that self-generated (subjective) narratives of high valence may counteract explicit CS–US valence information (e.g., Bar-Anan and Moran, 2017, p. 10). We illustrate this idea in Figure 1, where we describe how the proposition “eating is pleasant” may fail to augment actual eating. We posit that mere awareness of the word “eating” (CS) evokes the self-generation of a negatively valenced narrative associated with the CS representation (*eating*). These narratives can function to negatively valence the derived *eating* representations (e.g., to the effect “eating will make me fat and ugly and therefore I will become unattractive and lonely”), overriding any positively valences projected from the explicit *eating-pleasant* (CS–US) information. Our hypothesis explains how the proposition “eating is pleasant” may even induce reduced eating in certain individuals (i.e., those who tend to self-generate excessively negatively valenced narratives about eating; Merwin et al., 2010).

A representative study by Hensels and Baines (2016) provides evidence for our hypothesis. In that study, images of food (CS) were linked with happy or angry faces (US) during a CS–US conditioning task. After conditioning, participants evaluated CS via implicit association tests (IATs; Greenwald and Nosek, 2001) and decision-making tasks to determine, first, whether the valences for the food CS had transformed in accordance with their linked US and, second, whether the direction of the valence transformations (i.e., positive versus negative) influenced the motivation to consume CS-associated foods. Hensels and Baines found that subjects who were less likely to engage in emotional thinking were more receptive to external CS–US valence information, where evaluations toward CS foods shifted predictably (i.e., linking certain foods with happy faces led toward increased preferences toward those foods). Alternatively, subjects more likely to engage in emotional thinking were less susceptible to explicit valence information (CS–US pairings produced no significant effects on food preferences). These results showcase how subjective narratives can counter explicit CS–US valence information. It also may explain why strategies to counteract pathological behavioral patterns through talking strategies may not always alter the pattern in question (e.g., telling an anorexic person that “eating is good” and seeing no effect – again, see Merwin et al., 2010).

In the present study, we aimed at minimizing potential interference from CS-evoked narratives during CS–US conditioning by presenting CS too briefly (subliminally) to permit conscious identification (Custers and Aarts, 2010; Aarts and Custers, 2012). We assumed that narrative generation requires lexical processing of a CS representation. We also assumed that CS representations take longer to be redintegrated when based on partial visual information (Kouider et al., 2010). Briefly, “redintegration” describes how a significate becomes reinstated “as a memory or idea” in response to the presentation of a “partial constituent” of said significate (Warren, 2018). The notion resembles “feature integration” concepts across contemporary binding accounts of S-S learning, but without any a priori requirements for selective attention processes (Walther et al., 2018). A redintegrated CS representation may evoke valence responses prior to additional processing. By preventing conscious awareness of CS and their associated narratives, we hoped to enhance the probability of visible US directly transforming the redintegrated CS representations. That is, reducing the chance of seeing the verb “eating” should mitigate the onset eating-associated narratives. We addressed two questions in our present study. First, we asked whether US-to-CS valence transformations could be detected following the presentation of visible US and subliminal CS using magnetoencephalographic (MEG) source imaging. Next, we explored whether augmenting the valences of eating-associated CS would correlate with salivary, performance-based, and orally reported measures of appetite motivation.

We decided on stimulus-evoked MEG activations as our exclusive measure of valence for two reasons. First, assuming that awareness of a CS could suffice for a subject to generate a valenced CS-associated narrative, *any* behavioral measure of CS valence, whether explicit/implicit, incurs the possibility of CS-evoked narratives influencing evaluations (Gawronski and Hahn, 2018; March et al., 2018). By restricting our valence measurements to CS- and US-evoked electrophysiological components and never having subjects evaluate CS behaviorally, we minimized the possibility of CS-evoked narratives moderating our measurement of CS valence (Amd et al., 2013).

In spite of such precautions, it can still be the case that *some* ongoing narrative can influence CS valence expression. To increase the likelihood that we were assessing stimulus-evoked (as opposed to narrative-associated) valences, it is required to identify stimulus valence effects before lexical processing begins (typically within ∼200 ms of stimulus onset – Barber and Kutas, 2007). MEG and EEG provide the millisecond temporal resolution. MEG has the added advantage that magnetic brain signals do not get distorted by head tissues, which significantly enhance the topographical interpretation and source modeling of brain responses (Baillet, 2017). The combination of high spatial and temporal resolution is the second reason for selecting MEG to assess stimulus valence.

Previous research has demonstrated valence-associated event-related components (ERCs) within 100–200 ms of stimulus onset using EEG, typically over regions associated with language processing and feature redintegration (Amd et al., 2013; Kuchinke et al., 2015; Blechert et al., 2016, p. 14; Bayer et al., 2017). As our CS and US were naturally occurring words, we predicted ERCs localized over language areas would discriminate between neutral and positive US within 200–300 ms of US onset. Observing similar ERCs discriminating neutral from positive CS would indicate US-to-CS valence transformations.

We focused on positive versus neutral contrasts for two reasons. First, earlier investigations on event-related effects between positive, negative, and neutral terms highlighted positive/negative versus neutral items as typically being the most discernible (Rozenkrants et al., 2008; Hinojosa et al., 2009; Amd et al., 2013; Kissler and Herbert, 2013). This is because early electrophysiological responses are driven by overall stimulus salience, and positive/negative terms are typically more salient than neutral items (Olofsson et al., 2008; Hofmann et al., 2009). Another reason for employing only positive and neutral US is because we do not know about any long-term effects following the current procedure, hence it could be unethical to employ negative US. Specifically, if our present hypotheses are valid and appetite motivations can be subliminally influenced, employing negative US could pose the risk of disrupting normative eating patterns across otherwise healthy individuals.

Modulations of brain rhythmic fluctuations in the 8–12 Hz alpha frequency band also correlate with manipulations of stimulus valence (Simons et al., 2003; Aftanas et al., 2004; Uusberg et al., 2013; Amd and Roche, 2016, 2017; Marshall et al., 2018). Alpha oscillations may reflect inhibition in anticipation to salient events, where more salient stimuli are predicted to invoke greater selective cortical inhibition (augmented alpha power) over task-irrelevant regions (Onoda et al., 2007; Klimesch, 2012). Note that alpha modulations are not associated exclusively with stimulus valence and can be observed across multiple tasks that manipulate response inhibition (e.g., action observation versus action execution – Babiloni et al., 2016). We predicted that positively valenced US would induce greater alpha activity relative to neutral US. Similar alpha activations in the presence of CS would provide additional evidence of US-to-CS valence transformations. Since our task involved natural words, valence-associated ERCs and alpha activations were predicted over left central-parietal-temporal regions, which are key nodes of a distributed network for semantic (valence) comprehension (Binder, 2017; also see Brownsett and Wise, 2010; Meyer et al., 2013).

We employed three measures of appetite motivation in our study. First, after each conditioning trial, subjects had to answer Yes/No to the question “Are you getting hungry/sleepy?”. Our second (saliva) and third (oral) measures were implemented between blocks of conditioning trials. At the end of each block, we placed dental rolls in the subjects’ mouths to measure salivary volume. We predicted that increased saliva would follow conditioning trials where eating-related CS were linked with positive US, since increased saliva production is positively correlated with greater appetite motivation (Nirenberg and Miller, 1982; Epstein et al., 1996). After saliva was collected, subjects orally reported their preferences toward various activities (e.g., to the question “how much would you like to *run* right now?” presented on a screen). Some of the displayed activities functioned as CS during conditioning. We asked subjects to verbally respond to a visual prompt with minimal time/movement restrictions to maximize the probability our subject would deliberate (i.e., generate narratives) before responding. Assuming self-generated narratives can counteract explicitly provided valence information (Figure 1), we predicted that orally provided ratings would be least sensitive to our procedural manipulations, relative to all other measures of appetite motivation. We also included a measure of CS visibility after each conditioning trial to determine whether conscious CS identification is necessary for valence acquisition (Heycke and Stahl, 2018).

## Materials and Methods

### Subjects

Six males and seven females were recruited for the present study through personal invitation. Inclusion criteria for the study included normal/corrected-to-normal vision, proficiency in English, age (above 18), and no confounding medical histories (e.g., legal/illegal drug use, pre-existing medical conditions like schizophrenia). All subjects provided informed and written consent prior to participating. The data of two females were excluded as they turned out to be novel English speakers who were unfamiliar with nearly three quarters of the stimuli used; a third female was discarded due to extensive artifacts caused by her dental fillings. This left a final sample of *n* = 10 (27.5 ± 9.5 years). Seven of our 10 subjects had never undergone a MEG study previously, and nine among those 10 reported having never participated in psychological research. Everyone received CAD $50.00 for their time and were instructed not to eat two hours prior to the session. We did not want our subjects to arrive to the experiment satiated, which would reduce the probability of augmenting appetite motivations even if our eating-related CS had been positively valenced (Booth and Toase, 1983). All subjects were informed during the study’s onset that they were to commence a task measuring “attention,” with specific instructions to identify the CS word that appeared after the fixation. All subjects were fully de-briefed at the end of the study. The procedures were approved by the Research Ethics Boards at the McGill University Health Center and Montreal Neurological Institute (Approval # 2018-4166) and correspond with the guidelines provided in the Declaration of Helsinki. The duration of experimental sessions, including MEG setup and debriefing, was 90–120 min per subject.

### Materials

Stimuli designated to be CS included eight sleeping-related (resting, yawning, relaxing, slumbering, napping, snoring, dreaming, snoozing) and eight eating-related (snacking, nibbling, munching, chewing, consuming, devouring, gobbling, feasting) verbs. Stimuli designated to be US included eight positively valenced attributes (happy, rich, nice, pleasant, lovely, wonderful, enjoyable, great) and eight neutral nouns (chime, gray, door, building, lamp, bell, wall, window) terms. For positive and neutral US, the mean ± SD valences reported by Warriner et al. (2013) were 7.4 (±1.8) and 5.4 (±1.4) respectively (see Table 1 for arousal and dominance scores). These were corroborated in a separate investigation by the authors, where 15 subjects produced valences of 7.8 (±1.2) and 5.4 (±0.8) for the positive and neutral US, respectively. All ratings reflect scores on scales ranging from 1 (sad) to 10 (happy). The mean number of characters for positive and neutral US words was not significantly different (*p* = 0.156). US were not controlled along lexical frequency, imageability, and other topographical characteristics as these do not significantly mitigate subliminal conditioning (Greenwald and De Houwer, 2017). We also included “distractor” verbs (CS-) that were not used during conditioning trials (running, reading, dancing, writing, talking, swimming, jogging, speaking). Distractors were used during the two-alternative-forced choice (2AFC) tasks and between-condition oral sessions (see section “Procedure”). All CS were sandwiched by masks constituting of three X’ as these were not semantically relatable with our CS/US (Jaśkowski and Przekoracka-Krawczyk, 2005). Masks were of Arial black size 34 font. CS and US appeared in regular Arial size 22 font. Characters of size 34 and 22 fonts have em heights of 0.85 and 0.55 in each (“em” is a unit of measurement in typography). Corresponding em widths were 0.78 and 0.35 in, respectively. The total area spanned by three 34 font X’s equaled (0.85^∗^3^∗^0.78) = 1.99 square inches. The maximum area covered by a US (the longest of which had 11 characters) was (0.55^∗^11^∗^0.35) = 2.12 square inches. Despite the different number of characters, the maximal visual discrepancy between the masks and stimuli was less than 0.13 square inches. We assessed subjects’ ability to identify words at this distance by having them loudly read off unrelated words presented in a smaller font prior to the beginning of the conditioning task. All stimulus presentations were projected onto a screen from a video projector with a 60-Hz refresh rate. All statistical analyses were conducted on the R platform (R Core Team, 2014). All MEG pre-processing and analyses were completed with Brainstorm (Tadel et al., 2011) following guidelines for group analysis (Tadel et al., 2019).

**Table 1:** Mean ± SD of US valence, arousal, and dominance.

Condition^∗^	ID^∗∗^	Word	Valence	Arousal	Dominance
Positive	5596	Happy	8.5 ± 1.3	6.1 ± 2.1	7.2 ± 2.0
Positive	10380	Rich	6.8 ± 2.3	6.8 ± 2.0	6.8 ± 2.4
Positive	8178	Nice	7.0 ± 2.0	3.5 ± 2.4	6.5 ± 2.0
Positive	9153	Pleasant	7.2 ± 1.5	2.9 ± 2.5	6.7 ± 2.0
Positive	7250	Lovely	7.6 ± 1.7	4.0 ± 2.8	6.4 ± 2.4
Positive	13793	Wonderful	7.4 ± 1.8	4.6 ± 2.5	7.2 ± 2.1
Positive	4121	Enjoyable	7.6 ± 1.5	4.8 ± 2.2	7.0 ± 1.9
Positive	5375	Great	7.5 ± 1.9	4.1 ± 2.7	6.7 ± 1.8
		*Average*	*7.4 ± 1.8*	*4.6 ± 2.4*	*6.8 ± 2.1*
Neutral	2042	Chime	6.1 ± 1.2	4.1 ± 2.2	6.2 ± 2.2
Neutral	5369	Gray	3.7 ± 1.7	2.8 ± 1.9	4.8 ± 2.2
Neutral	3727	Door	5.4 ± 1.2	3.2 ± 2.0	6.1 ± 2.1
Neutral	1570	Building	5.5 ± 1.1	3.4 ± 2.2	6.0 ± 2.2
Neutral	6904	Lamp	5.7 ± 1.1	2.7 ± 1.8	6.0 ± 2.2
Neutral	13532	Wall	5.1 ± 1.7	2.9 ± 1.9	4.2 ± 1.7
Neutral	13738	Window	6.5 ± 1.6	3.3 ± 1.9	5.4 ± 2.6
Neutral	1054	Bell	5.7 ± 1.8	4.7 ± 2.3	6.2 ± 2.4
		*Average*	*5.4 ± 1.4*	*3.4 ± 2.0*	*5.6 ± 2.2*


*^∗^Neutral and positive terms appeared as USs across separate blocks. ^∗∗^IDs refer to the dataset from Warriner et al. (2013).*

### Procedure

#### Overview

Subjects were fitted with non-magnetic scrubs upon arrival and comfortably seated in an upright position under the MEG helmet. The experimenter provided the subject with two sterile dental rolls to place in their mouth. After a minute, the rolls were collected and weighed within 10 s. The difference in dental roll weights before and after being placed in the subject’s mouth was recorded as salivary volume. Next, subjects viewed prompts on the computer screen to orally call out a rating between 1 and 10 in response to the questions “How much would you like to (activity) right now? (Where ‘1’ means ‘not at all’ and ‘10’ means ‘a lot’)” presented in no specific sequence. Subjects provided preferences for four activities, two of which resembled our eating- and sleeping-related CS.

**FIGURE 2:**
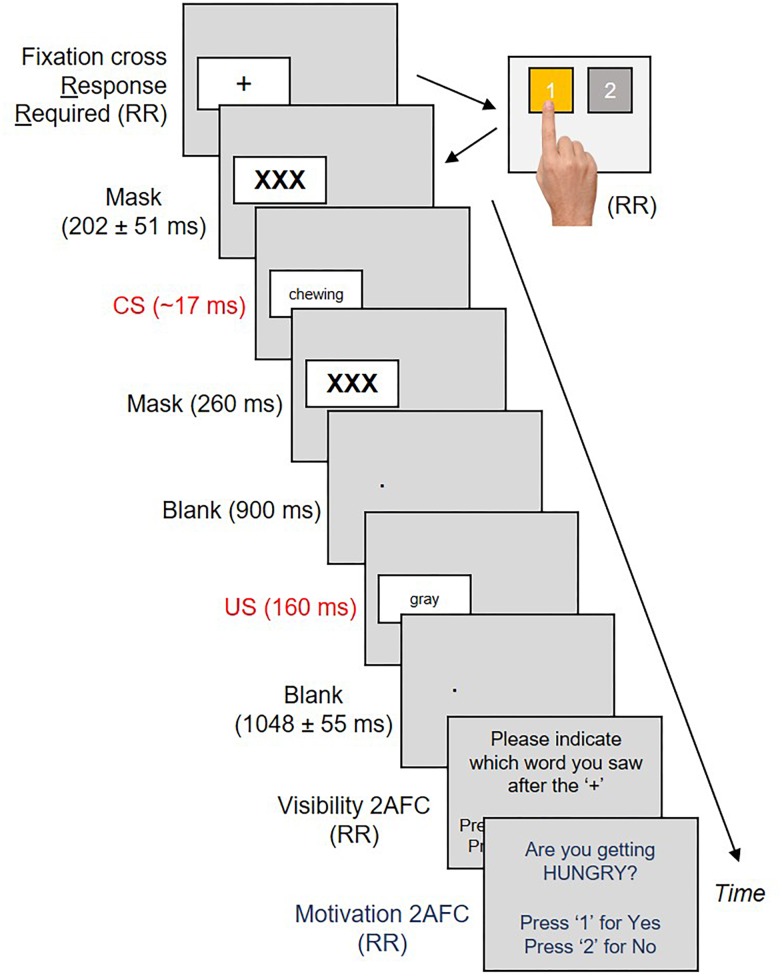
Each conditioning trial commenced with a fixation cross on the left or right sides of the screen. Subjects had to respond to the location of the cross by pressing “1” or “2” on a button box. This produced a CS sandwiched between masks, a blank interval and a US in the same location as the cross. US offsets produced a second blank interval, followed by two two-alternative forced choice tasks measuring CS visibility and motivational state, respectively.

Salivary volume and oral preferences were collected five times for each subject over the course of the experiment; at baseline before the procedure began, and once after each of the four conditioning blocks. Across two of these blocks, CS were exclusively linked with neutral US. Across the two remaining blocks, CS were exclusively linked with positive US. The sequence of neutral (N) and positive (P) trial blocks were counter-balanced across participants, so that half our subjects underwent an N→P→N→P sequence, whereas the remaining half underwent a P→N→P→N sequence. Each block contained 80 conditioning trials. Each conditioning trial commenced with a black fixation cross inside a white box on the left or right sides of the screen. Subjects had to produce a button press corresponding to the left/right position of the cross (Amd et al., 2017). An accurate button-press (1 = Left, 2 = Right – see Figure 2) produced a jittered forward mask for 202 ± 51 ms, followed by an eating/sleeping related verb (CS+) for ∼17 ms and a backward mask for 260 ms. This was followed by a second blank screen for 900 ms with a white fixation point in place of the box that had appeared earlier. The box next re-appeared in the same location with a US for 160 ms. Our US remained visible in order to reliably evoke valenced responses (Lähteenmäki et al., 2015). The US was replaced with a second blank screen (1048 ± 55 ms) and our first 2AFC (visibility check – Figure 2). Subjects had to select from two options (CS+/CS-) which word they thought had appeared earlier. A response produced a second 2AFC, where subjects had to respond Yes/No to the question “Are you getting hungry (sleepy)?”. A response here initiated a third blank screen for 500 ms, marking the end of that trial.

#### MEG Acquisition and Pre-processing

Magnetoencephalographic activity was recorded using a 275-channel whole-head MEG system (CTF/VMS, BC, Canada) with a sampling rate of 2400 Hz and a 0–150 Hz filter bandwidth. We measured vertical (VEOG) and horizontal (HEOG) eye movements by placing two electrodes ∼1 in above and below the right eyeball, and two electrodes near the left and right temporal bones. Cardiac activity (ECG) was recorded by placing one electrode near the second interspace left midclavicular line, and a second electrode near the eighth interspace midclavicular line. VEOG, HEOG, and ECG activity was recorded to inform artifact correction during analysis. Head position was determined by placing three head localization coils at three fiducial points (nasion, left ear, right ear). Anatomical registration with the default MRI anatomy (Colin27_2016 template; Brainstorm) was facilitated with the manual addition of ∼120 digitized points using a 3-D Polhemus Isotrack digitizer system along the top surface of the head and nose for all our subjects. Head position was continuously recorded with a sampling rate of 150 Hz to ensure that subject head movements did not exceed 1 cm during and between conditioning trials. CS and US onset durations were confirmed to fall within ±1 ms of 16.6 and 160 ms, respectively, through timing information provided by photodiodes. The photodiodes were not visible to subjects during stimulus presentations.

We pre-processed our data according to the recommendations of Gross et al. (2013). All data were visually inspected for head movement and environmental noise artifacts, which were corrected during analysis through the use of signal-space projectors (SSPs; Tadel et al., 2011). A notch filter of 60 Hz was applied to remove powerline contamination. Heart and eye movement artifacts were detected from the EOG and ECG electrodes, respectively. We calculated SSPs from segments of data centered around the artifacts of interest. SSPs were defined following principal component analysis of artifact-contaminated segments, filtered between 10–40 and 1–15 Hz for heartbeats (150 ms segment duration) and eye-blinks (400 ms segment duration), respectively. We rejected 13/1600 and 81/1600 trials from the neutral and positive conditions, respectively, which allowed us to retain >95% of the collected data. We bandpass filtered our data between 0.5–1 (high-pass) and 40–46 Hz (low-pass) using a linear phase finite impulse response filter with stopband attenuation at 60 dB. We down-sampled our data to 400 Hz for analysis, which retained a frequency resolution of 0.012 Hz. We epoched our data into 2400-ms windows [-200, 2200 ms], centered around CS [0 ms] and US [1170 ms] onsets, for analysis of ERCs and alpha power.

#### Source Estimation

**FIGURE 3:**
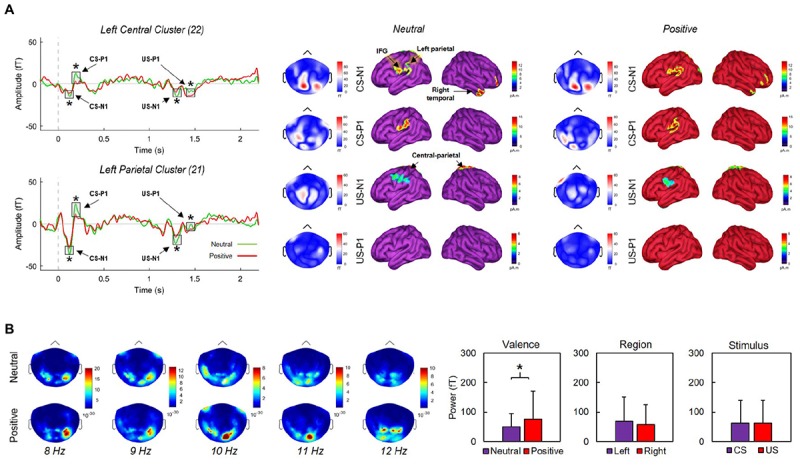
**(A)** Grand averaged activity over left central and parietal clusters over a 2400 ms window (left) and corresponding source activations during neutral (middle) and positive (right) blocks. CS and US onsets were at 0 and 1170 ms, respectively. Early negative (N1) and positive (P1) event-related components produced by CS and US onsets differed significantly between neutral and positive conditions (asterisks indicate *p*’s < 0.02) across the displayed clusters. **(B)** Mean activation maps across five 1-Hz bands corresponding to the alpha range. Alpha power did not vary significantly between left and right regions, or between stimulus types. Significantly greater alpha (*p* = 0.001) appeared during positive conditions only. Error bars indicate SDs.

We produced a MEG head model to estimate the cortical sources underlying the magnetic fields detected by the sensors during recording. The forward model was computed from a default cortical surface representation with 15,000 vertices using the overlapping-spheres analytical method (Huang et al., 1999). We derived MEG source maps from the weighted minimum-norm estimate available in Brainstorm using default parameters (Baillet et al., 2001). Noise covariance statistics were derived from a 200-ms pre-CS [-200, 0 ms] baseline taken across trials. To account for inter-subject variability, the orientation of elementary cortical current dipoles was not constrained to the cortical surface template. Source maps were produced from trial averages for each condition (neutral X positive). Figure 3 illustrates cortical activations following CS/US onsets.

## Results

### MEG Correlates of US-to-CS Valence Transformations

#### ERCs

We first defined sensor clusters of interest over ten cortical sites. These included left (LF = 32 sensors) and right frontal (RF = 32), parietal (LP = 21, RP = 21), central (LC = 22, RC = 22), temporal (LT = 32, RT = 32), and occipital (LO = 18, RO = 18) sites. Second, we extracted absolute peak/trough amplitudes across each cluster along epochs. Third, we identified N1 and P1 components within 80–130 (CS-N1) and 170–220 ms (CS-P1) of CS onset, and within 90–140 (US-N1) and 200–250 ms (US-P1) of US onset, following inspection of ERC topographies over parietal regions. Finally, we contrasted each component between neutral and positive conditions using Welch’s *t*-tests. We found significant effects for ERCs over left-central (LC) and left-parietal (LP) clusters (Figure 3). Over the LP cluster, N1 components were significantly more negative-going during neutral conditions for CS, *t*(30.8) = 3.97, *p* < 0.001, *d* = -1.22, and US, *t*(38.9) = 5, *p* < 0.001, *d* = -1.54; P1 components were significantly more positive-going during neutral conditions for CS, *t*(32.6) = 12.53, *p* < 0.001, *d* = 3.87, and US, *t*(36.8) = 14.83, *p* < 0.001, *d* = 4.58. Similar modulations were observed over the LC cluster; N1 components for CS, *t*(34.7) = 2.56, *p* = 0.015, *d* = -0.79, and US, *t*(39.9) = 3.21, *p* = 0.003, *d* = -0.99, were significantly more negative-going during neutral conditions. P1 components for CS, *t*(32.2) = 13.45, *p* < 0.001, *d* = 4.15, and US, *t*(32.9) = 8.24, *p* < 0.001, *d* = 2.54, were significantly more positive-going during neutral conditions. Finally, we found significant N1/P1 effects over our right parietal cluster following US onsets (all *p*’s < 0.001), but not CS onsets (all *p*’s > 0.05). None of our remaining contrasts reached significance.

#### Alpha Oscillations

We computed power spectral densities (PSDs) over 2400-ms windows (-200 to 2200 ms where CS onset was at 0 ms) using Welch’s method with a 1000-ms sliding window and 50% overlap along and across individual trials from each condition (positive X neutral). Cortical maps illustrating the location of peak alpha power across individually defined bands between 8 and 12 Hz are presented in Figure 3. We extracted PSDs in the alpha band from sensor time series over 0–1000 and 1000–2000 ms windows, corresponding with CS and US onsets, respectively. The 40 selected sensors were located over the left (20) and right (20) parietal sites. We ran three Welch’s *t*-tests to determine whether PSDs significantly differed as a function of stimulus type (CS, US), region (left, right), and/or valence (neutral, positive). We found no significant effects for region (*p* = 0.237) or stimulus-type (*p* = 1). For valence, PSDs during positive conditions (76.3 ± 95.7) were significantly greater, *t*(236.4) = 3.22, *p* = 0.001, *d* = 0.35, than PSDs during neutral conditions (50.1 ± 44.6). Conducting similar contrasts over other traditionally defined frequency bands (beta, theta, gamma) produced no significant differences between conditions (all *p*’s > 0.05).

### Measures of Appetite Motivation

#### Salivary Volume

**FIGURE 4:**
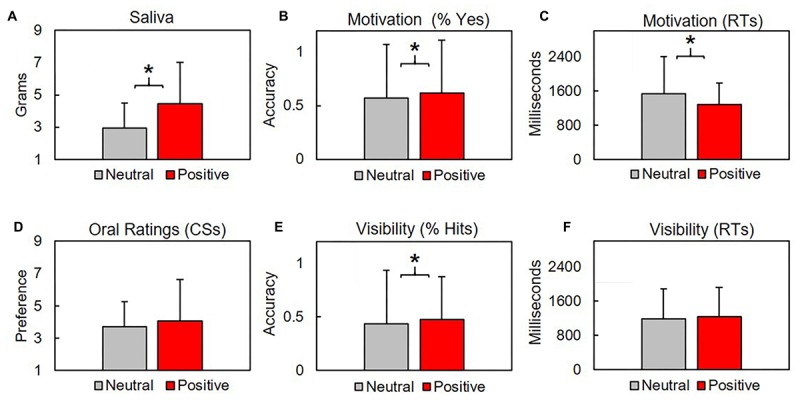
Subjects produced significantly more saliva after positive conditioning blocks **(A)**. Subjects responded *Yes* significantly more times **(B)** and faster **(C)** in response to the question “Are you getting hungry?” during positive conditioning trials. Oral preferences did not differ between conditions for CS **(D)** or distractors (not shown), where higher values indicate increased preference (*y*-axis). CS were identified significantly more times during positive conditioning trials **(E)**, although hit rates across both conditions fell below chance levels. Response times for visibility checks did not vary across conditions **(F)**. Asterisk indicate *p*’s < 0.03.

We contrasted mean ± SD grams of saliva produced after CS were linked with neutral US (2.96 ± 1.54 g) with saliva produced after CS were linked with positive US (4.47 ± 2.54). Subjects produced significantly more saliva after positive conditioning blocks, *t*(31.3) = 2.27, *p* = 0.03, *d* = 0.72 (Figure 4). Saliva produced by subjects before conditioning (5.7 ± 5.38) was not included in our analysis for two reasons: first, subjects provided a single pre-conditioning saliva sample, but four post-conditioning saliva samples (two neutral, two positive). Second, as the samples were collected before conditioning, they were unrelated to our procedural valence manipulations.

#### Oral Ratings

Subjects orally rated four activities at the end of each block. Two activities were related to our CS (eating, sleeping). Remaining activities were distractors (running, reading) that were not used during conditioning. Higher scores correspond with increased preference. CS preference between positive (4.08 ± 1.89) and neutral (3.7 ± 1.88) blocks did not significantly vary (*p* = 0.376). Preferences for distractors between positive (5.25 ± 0.38) and neutral (5.5 ± 2.57) blocks did not vary (*p* = 0.655). See Figure 4.

#### Motivation Check

Subjects responded Yes/No to the questions “Are you getting hungrier (sleepier)?” after each conditioning trial. We found the mean proportion of Yes responses between positive (0.62 ± 0.49) and neutral (0.57 ± 0.5) blocks was significantly different, *t*(2553.2) = 2.48, *p* = 0.013, *d* = 0.09. Response times (RTs) during positive (1274 ± 667.5 ms) and neutral (1531.1 ± 865.9) significantly differed, *t*(2155.5) = 8.62, *p* < 0.001, *d* = 0.34. See Figure 4.

### CS Visibility

Subjects selected from two verbs during the visibility check 2AFC across each trial. A response was scored as a (correct) hit if subjects accurately identified the verb that had previously appeared as a CS. Subjects produced significantly more hits during (0.47 ± 0.5) relative to neutral (0.43 ± 0.5) conditions, *t*(2594.7) = 2.08, *p* = 0.038, *d* = 0.079, although the number of correct detections across both conditions was lower than would be expected by random chance (0.5). RTs did not significantly differ between neutral (1188.8 ± 691.6 ms) and positive (1231.7 ± 692.7 ms) conditions (*p* = 0.101). See Figure 4.

## Discussion

Our study linked eating- and sleeping-related CS with neutral and positively valenced US across four blocks of CS-US conditioning trials. The CS were presented subliminally and not reliably identified beyond chance levels. Analysis of time-resolved electrophysiological components showed significant valence-associated disassociations of stimulus-evoked cortical activations within 200 ms of CS onset, i.e., before the expected engagement of lexical processing. These disassociations were significant over left parietal and central regions of interest, which are involved in lexical-semantic comprehension (Binder, 2017). We found alpha activity over parietal regions to be significantly more pronounced during positive conditioning trials. Finally, we observed greater production of saliva and Yes responses (to the questions *Are you getting hungrier/sleepier?*) following blocks of positive conditioning trials. Only orally provided preferences did not vary between conditions.

Our results show that subliminally enhanced CS valences can augment CS-associated motivational states, as previously claimed by Aarts and Custers (2012). Our study is the first to demonstrate the disassociation of valence-associated ERCs following the presentation of *subliminal* CS and supraliminal US within pre-lexical windows. We claim that the absence of effect across our oral reports actually supports our initial hypothesis. Indeed, our oral preferences task was designed to be biased by self-narratives: they maximized the possibility of participants deliberating (generating narratives) prior to responding. This interpretation is compatible with the observation that it took ∼4 s between presentation of a question during the oral ratings task (*How hungry are you?* for example) and the contingent response. For comparison, our second behavioral measure of motivation produced RTs under 2 s and was far less likely to have been driven extensively by deliberative processes. This illustrates how CS-associated narratives can mitigate explicit CS–US valence information.

Our framework posits that (i) motivational states, like hunger, can be significantly modulated through the construction and integration of self-generated emotionally salient narratives (Peterson, 2002; also see Van Vugt et al., 2018), (ii) the representational content from which self-narratives are constructed is a function of externally available relational information and earlier learning histories (Osgood, 1980; Bar-Anan and Moran, 2017; Fields and Arntzen, 2018), and (iii) external valence information can be encoded without a perceiver’s complete awareness of the valence-specifying stimulus/relation perceived (Kouider et al., 2010). We found physiological evidence in support of this hypothesis, given that observed physiological effects incorporated semantic and motor regions (Boulenger et al., 2008).

Our present findings corroborate embodied-cognition perspectives that propose how language comprehension relies on the internal reenactment of sensorimotor activations associated with a specific stimulus (Vigliocco et al., 2009). The embodiment of stimulus-associated response mechanisms resembles earlier neo-associationist accounts of symbolic behavior, where fractional stimulus-response (sG-rG) associations exclusive to a representational class were hypothesized to probabilistically mediate response tendencies through spreading activation mechanisms, such as those involving stimulus convergence, response divergence, and secondary generalization (Hull, 1930; Berlyne, 1965; Osgood, 1980).

These findings also provide a roadmap for possible future intervention strategies to address (for example) eating disorders resistant to explicit valence information, such as anorexia nervosa (Merwin et al., 2010). Taken together, our results suggest that urges to engage in pathological eating practices may be mitigated through preventing the conscious perception of eating-associated stimuli/cues (Custers and Aarts, 2010). Future research could extend these findings to subjects prone to “emotional eating” (Hensels and Baines, 2016) to determine whether subliminally augmenting the valences of eating representations motivates actual eating, and whether such motivations may be transitory or cumulative (e.g., Mattavelli et al., 2017). If the latter, the logical next step would be to adapt the proposed procedure for use with anorexic individuals, whose maladaptive belief systems regarding eating constitutes a core element of their pathology (Merwin et al., 2010).

We conclude our discussion by noting some potential limitations of the current design. First, neutral and positive US appeared in separate blocks instead of being mixed within the same block. In fact, US were always visible to subjects, whereas CS were visible only in 50% of trials or less. We reasoned that if positive and neutral US had appeared during the same block of trials, and these were the only items subjects could consciously identify, then their simple co-occurrence (e.g., *gray* with *pleasant*) could have consequated the unprovoked derivation of a relational qualifier between the differentially valenced terms (e.g., *gray* “co-occurs with” *pleasant* – De Houwer, 2018, p. 5). In other words, neutral US could have transformed into valenced CS. By separating US across blocks, any potential US-to-US valence “contamination” effects were mitigated (Pastor et al., 2015). Future investigations could nevertheless use a mixed design to determine whether neutral US remain neutral. A second concern may be raised regarding the relatively small sample size (*n* = 10). In response, we point out that our ERC effects were quite robust, replicating earlier EEG findings (Amd et al., 2013; Bayer et al., 2017). We also report large effects for our saliva and performance-based motivation checks. The small sample size nevertheless is an important limitation that requires us to be cautious regarding any substantial interpretations – future replication studies should employ larger samples to determine the generality of the present findings.

One criticism may be our present focus on central-parietal ERCs, since earlier works have demonstrated valence-specific ERCs over frontal motor regions following the presentation of action words, although at time windows too late to reflect pre-lexical processes (Aarts et al., 2013, p. 969). Additionally, our US were non-action terms and thus not predicted to engage motor regions differentially during pre-lexical windows. Given that frontal/pre-frontal regions engaged by verb representations also employ central-parietal networks during pre-lexical windows (Hauk and Pulvermüller, 2004, p. 197), future works could employ valenced action words as US to determine whether pre-lexical differences appear over frontal motor regions (e.g., M1, BA6). This would illustrate whether the sensory-motor/fractional response components associated with activities may be augmented independent of their associated significate.

Another criticism may be leveled at our ascription of valence to CS given that subliminally presented stimuli do not reliably evoke valences isomorphic with the functions implied by the actual stimulus topographies/features (Kouider et al., 2010). For those concerned with that label, imagine “CS valence” as a summary description of the affective response components associated with redintegrated pre-lexical representations evoked by CS onsets. In support of the notion that our CS representations were *pre*-lexical, note that our examined effects appeared within 200 ms of stimulus onset, whereas lexical processing onsets within ∼250 ms of stimulus perception (Schacht and Sommer, 2009; Palazova et al., 2011). Fragments of a percept may redintegrate into meaningful representations based on partial visual information, even if the reconstructed representations do not structurally cohere with the actual stimuli they represent (Kouider et al., 2010; Tonneau, 2013). Indeed, the time courses of our ERCs provide compelling evidence that bottom-up stimulus valence produced the observed effects (Kissler and Herbert, 2013).

A final issue can be raised regarding our interpretation of early ERCs as indicative of stimulus valence instead of, say, the engagement of attentional resources (Vogel and Luck, 2000). In contrast, we propose that *orienting* (non-volitional attending – see Maltzman, 1979; Amd et al., 2017) constitutes the initial affective discrimination toward valence gradients embedded in perceived stimulus objects, however minute such gradients may be (Lebrecht et al., 2012). From this perspective, early ERCs thought to reflect “selective attention, object recognition, and categorization” (Blechert et al., 2016, p. 16) are conceptualized as anticipatory response links pre-empting stimulus redintegration (Berlyne, 1965; Osgood, 1980). Specifically, orienting is the first non-volitional emotional response anteceding the start of a contextualized stimulus-response chain (Hull, 1930). The notion of a S-R chain anticipated by a valenced representation allows the theoretical separation of stimulus-evoked and narrative-associated valences. This is supported by the early time window of our pre-lexical ERCs, since higher order processing vis-à-vis attention/recognition/categorization takes more processing time and resources.

Regardless of one’s preferred theoretical flavor, a majority of perspectives agree that the integration of self-generated and explicitly-provided valence representations manifest in actual, experienced valence (Peterson, 2002). When representations are action-oriented (as evoked by the verb “eating”), their evocations generalize to response mechanisms associated with said action (increased saliva production). Our study demonstrates how subliminally augmenting the valences of eating-related representations can significantly moderate associated motivational states.

## Author Contributions

MA designed the study and collected and analyzed the data. Both authors contributed toward the writing of the manuscript.

## Conflict of Interest Statement

The authors declare that the research was conducted in the absence of any commercial or financial relationships that could be construed as a potential conflict of interest.
